# Global Characteristics of CSIG-Associated Gene Expression Changes in Human HEK293 Cells and the Implications for CSIG Regulating Cell Proliferation and Senescence

**DOI:** 10.3389/fendo.2015.00069

**Published:** 2015-05-15

**Authors:** Liwei Ma, Wenting Zhao, Feng Zhu, Fuwen Yuan, Nan Xie, Tingting Li, Pingzhang Wang, Tanjun Tong

**Affiliations:** ^1^Research Center on Aging. Department of Biochemistry and Molecular Biology, School of Basic Medical Sciences, Peking University Health Science Center, Beijing Key Laboratory of Protein Posttranslational Modifications and Cell Function, Beijing, China

**Keywords:** CSIG/RSL1D1, senescence, cell cycle, gene expression, microarray

## Abstract

Cellular senescence-inhibited gene (CSIG), also named as ribosomal_L1 domain-containing 1 (RSL1D1), is implicated in various processes including cell cycle regulation, cellular senescence, apoptosis, and tumor metastasis. However, little is known about the regulatory mechanism underlying its functions. To screen important targets and signaling pathways modulated by CSIG, we compared the gene expression profiles in CSIG-silencing and control HEK293 cells using Affymetrix microarray Human Genome U133 Plus 2.0 GeneChips. A total of 590 genes displayed statistically significant expression changes, with 279 genes up-regulated and 311 down-regulated, respectively. These genes are involved in a broad array of biological processes, mainly in transcriptional regulation, cell cycle, signal transduction, oxidation reduction, development, and cell adhesion. The differential expression of genes such as ZNF616, KPNA5, and MAP3K3 was further validated by real-time PCR and western blot analysis. Furthermore, we investigated the correlated expression patterns of Cdc14B, ESCO1, KPNA5, MAP3K3, and CSIG during cell cycle and senescence progression, which imply the important pathways CSIG regulating cell cycle and senescence. The mechanism study showed that CSIG modulated the mRNA half-life of Cdc14B, CASP7, and CREBL2. This study shows that expression profiling can be used to identify genes that are transcriptionally or post-transcriptionally modified following CSIG knockdown and to reveal the molecular mechanism of cell proliferation and senescence regulated by CSIG.

## Introduction

Cellular senescence, a natural barrier to cancer progression, is causally implicated in generating age-related phenotype (1–5), but the fundamental mechanisms that drive senescence remain largely unknown. Using a suppressive subtractive hybridization, we have identified and cloned a cellular senescence-inhibited gene (CSIG) (GenBank accession No. AY154473, http://www.ncbi.nlm.nih.gov) (6). CSIG is a Ribosomal_L1 Domain-Containing Protein and therefore was also named as RSL1D1 in the Human Genome Organization (HUGO) Nomenclature Committee Database. CSIG is abundantly expressed in early-passage fibroblasts, but its expression declines during cellular senescence. CSIG modulated cell cycle progression, in turn promoting cell proliferation (7). Moreover, overexpression of CSIG significantly delayed the progression of replicative senescence, while knockdown of CSIG expression accelerated replicative senescence (7). Our findings indicate that CSIG acts as a novel regulatory component of replicative senescence. Consistently, Meng et al. and Zhu et al. reported that CSIG/RSL1D1 could regulate the activity of nucleostemin which delays the aging progression in mouse fibroblasts (8, 9). In addition, Li et al. found that CSIG is required for p33ING1 to induce apoptosis under UV irradiation (10). Moreover, emerging evidences have indicated that CSIG might implicate in various biological processes such as breast cancer metastasis (11), tumor cell survival (12), inflammation (13), and bone formation (14).

According to informatics analysis (available at http://www.expasy.org), CSIG is evolutionarily conserved and human CSIG protein contains part of Ribosomal L1p/L10e consensus sequence (residues 30–260) in the N-terminus and a long Lys_rich domain (residues 280–485) in the C-terminus, suggesting that it may participate in ribosome biosynthesis or act as a transcriptional co-factor. Our previous studies have identified CSIG as a nucleolus protein accumulated in ribosome (7). Although the non-ribosomal functions of CSIG to regulate proliferation, apoptosis, and senescence progression have been established (7, 10); however, the molecular basis underlying is poorly understood.

In this investigation, we have compared differential gene expression patterns in HEK293 cells between CSIG knockdown and control samples using a fold change (FC) ≥1.5 as a cutoff to define CSIG-related gene expression and profile changes. We have demonstrated that gene expression changes associated with CSIG knockdown in 293 cells impact transcription regulation, cell cycle, development, and certain critical signal transduction pathways. Furthermore, we have identified candidate genes for further in depth analysis. Changes in expression patterns provide further evidence and a molecular basis for CSIG to regulate cell proliferation and senescence.

## Materials and Methods

### Cell culture

Human embryonic kidney cell (HEK293) was cultured in DMEM medium, containing 10% fetal bovine serum. Human diploid fibroblasts 2BS cell line from human female embryo lung was established at the National Institute of Biological Products (Beijing, China) and has been fully characterized (6, 7). The expected replicative life span of 2BS cells is about 70 population doublings (PDs). 2BS cells were considered to be young (early-passaged) at PD 30 or below and fully senescent at PD 55 or above. Senescent cells are characterized by an irreversible growth arrest and accumulated p16INK4a. 2BS cells were maintained in Dulbecco’s modified Eagle’s medium (Invitrogen) supplemented with 10% fetal bovine serum, 100 U/ml penicillin, and 100 μg/ml streptomycin, at 37°C in 5% CO_2_.

### CSIG knockdown in 293 cells/plasmids and transfection

The pcDNA3.1-CSIG and control vectors were purified with QIAGEN Plasmid Maxi Kits. Cells were transfected with plasmids coated by Lipofectamine 2000 (Invitrogen) following the manufacturer’s indications. To transiently silence CSIG, siRNA targeting CSIG (siCSIG) and control siRNA were synthesized (Genema), respectively. siRNAs were transfected with Lipofectamine 2000 (Invitrogen) following the manufacturer’s recommendations. Cells were collected 48 h after transfection for further analysis. The siRNA sequences were as follows:

- CSIG siRNA: 5′-AGAAGGAACAGACGCCAGA-3′
- Control siRNA: 5′-TTCTCCGAACGTGTCACGT-3′

### Western blotting

Cells were washed with PBS, collected, and lysed on ice for 30 min with RIPA (Applygen Technologies Inc., Beijing, China) containing a protease inhibitor mixture (Fermentas). Cell lysates were then centrifuged for 10 min at 15,000 × *g* at 4°C. The supernatant was collected, and the protein concentration was determined using the BCA Protein Assay Reagent (Pierce). Total protein (20 ~ 40 μg) was subjected to 10 ~ 15% sodium dodecyl sulfate-polyacrylamide gel electrophoresis (SDS-PAGE) and was transferred to nitrocellulose membranes (Millipore). After blocking in 5% non-fat dry milk in TBST (10 mm Tris-Cl, pH 7.5, 150 mm NaCl, 0.05% Tween 20), the membranes were incubated with primary antibodies overnight at 4°C. The membranes were then washed three times with TBST and then incubated with HRP-conjugated secondary antibodies (Zhongshan Biotechnologies Inc., China) for 1 h at room temperature. Proteins were visualized using chemiluminescent substrate (Millipore) according to the manufacturer’s instructions. Blots were probed with the following antibodies: anti-CSIG [used as previously described (7)], anti-p16 (sc-759, Santa Cruz), anti-ESCO1 (ab128312, Abcam), anti-Cdc14B (sc-374572, Santa Cruz), anti-KPNA5 (ab81450, Abcam), anti-MAP3K3 (ab40750, Abcam), anti-Cdc2 (E53, Epitomics), and anti-PCNA (BS1289, Bioworld).

### RNA extraction

Total RNA was isolated from HEK293 cells and 2BS cells using an RNeasy Mini kit (Qiagen) according to the manufacturer’s instructions. The quality of the RNA samples was examined by quantifying the A260:A280 ratio (the minimal acceptable ratio is 1.7) and the 28S/18S by visualizing rRNA bands in agarose gel (the minimal acceptable ratio is 1.5).

### Affymetrix cDNA microarray

The microarray screen was performed in triplicate using Affymetrix microarray Human Genome U133 Plus 2.0 chips containing 38,500 genes. Briefly, 15–20 g of biotin-labeled cRNA was fragmented by incubating in a buffer containing 200 mmol/l Tris acetate (pH8.1), 500 mmol/l KOAc, and 150 mmol/l MgOAc at 95°C for 35 min. The fragmented cDNA was hybridized with a pre-equilibrated Affymetrix chip at 45°C for 14–16 h. The hybridizations were washed in a fluidic station with non-stringent buffer (6× SSPE, 0.01% Tween 20, and 0.005% antifoam) for 10 cycles and stringent buffer (100 mmol/l 2N-morpholino-ethanesulfonic acid, 0.1M NaCl, and 0.01% Tween 20) for 4 cycles and stained with strepto-avidin phycoerythrin. This was followed by incubation with biotinylated mouse antiavidin antibody and restained with strepto-avidin phycoerythrin. The chips were scanned in an Agilent ChipScanner (Affymetrix Inc., Santa Clara, CA, USA) to detect hybridization signals.

Baseline analyses were done with AGCC to identify statistically significant gene expression alterations between samples derived from HEK293 cells transfected with siCSIG and siNC, respectively. Because samples were analyzed in triplicates, these results were additionally screened for consistent P by the Student’s *t*-tests (*P* < 0.05) to eliminate random sampling errors.

### Quantitative real-time PCR

Real-time PCR analysis was performed in triplicate using the SYBR Green PCR Master Mix (Applied Biosystems) on an ABI Prism 7300 sequence detector (Applied Biosystems). Each PCR was assembled using 96-well MicroAmp Optical plates (Applied Biosystems) with a total volume of 15 μl containing 1.5 μl cDNA templates, 1 μM of each primer, and 7.5 μl of 2× SYBR Green Master Mix and brought to final volume with RNase-free water. Thermal reaction cycles of 50°C for 2 min, 95°C for 10 min, and 40 repetitions of 95°C for 15 s and 60°C for 1 min were used. The data were analyzed using the ΔΔCT method, normalizing the *C*_t_ values of the indicated gene to the *C*_t_ values of GAPDH relative to a control sample. The GAPDH gene served as an endogenous control for normalization. Gene-specific primers were designed using Primer 5. The primer sequences used in this study are shown in **Table** 1.

**Table 1:** **DNA sequences of the primers used for quantitative real-time PCR**.

Identity/gene	Nucleotide sequences
ZNF367	Forward: 5′-AACCGCCACTGTCCGAAGCA-3′
	Reverse: 5′-CCTTTCAAAGTGGGGGTGCGCT-3′
ZNF616	Forward: 5′-TGGAAATGCCTGGAGCCTGTGC-3′
	Reverse: 5′-GGCCCGATGAAAGGCTTTGCCA-3′
KPNA5	Forward: 5′-GCAGACGTGTGTTGGGCCCTTT-3′
	Reverse: 5′-TCCATTGGTGCTTCCTGCTGCT-3′
CASP7	Forward: 5′-AAATGCCGCCTGCTTCGCCT-3′
	Reverse: 5′-TGGAGCAGAGGGCTTGCACA-3′
PPM1A	Forward: 5′-CGGCTGTGATCGGTTTGCCA-3′
	Reverse: 5′-GCCAGAGAGCCATTCACACGCT-3′
SETD7	Forward: 5′-TGAACGGTCCAGCCCAGGAA-3′
	Reverse: 5′-ACTGCTCTCAGGGTGCGGAT-3′
CREBL2	Forward: 5′-CGTGGTCGGAAGCCAGCCAAAA-3′
	Reverse: 5′-TTCGGGCTCGGCATTCTCTTGC-3′
NOLC1	Forward: 5′-AGCCCAAGGCGACTGCCAAA-3′
	Reverse: 5′-GCTGCCCCGCTTCTTCTTGGTT-3′
TRAK1	Forward: 5′-ACGGCAGCGACATAGGCAAC-3′
	Reverse: 5′-AGCAGAAATGCCCCGCTCCT-3′
CCDC115	Forward: 5′-CTGGAGGGGAAACGAACGGTGT-3′
	Reverse: 5′-ATGCGGTTCTGGAGGCTGGCTA-3′
C11orf24	Forward: 5′-TCAGCACAGCCCTCGCACAA-3′
	Reverse: 5′-ACCTTGTGCTTGGGGACGCA-3′
MTA2	Forward: 5′-AAGGAACGGCTACGACCTGGCT-3′
	Reverse: 5′-AACAGGAAGCACAGAGGCGGCA-3′
PCK1	Forward: 5′-AAGGTTGAGTGCGTCGGGGA-3′
	Reverse: 5′-TTCCCAGTAAACGCCCCCGT-3′
ESCO1	Forward: 5′-ACGAAACGAAACCTGTGCCTGT-3′
	Reverse: 5′-AGGCACTGATGGCTGTGGACT-3′
SEH1L	Forward: 5′-GCTCTCGTGCTCATTCCCCCAT-3′
	Reverse: 5′-GGCAGTGTCAGCATCGCAAGAGT-3′
RAB31	Forward: 5′-GGGGACACTGGGGTTGGGAAAT-3′
	Reverse: 5′-AGGTCGCACTTGTTTCCAGCG-3′
STAT1	Forward: 5′-TGGAGTGGAAGCGGAGACAGCA-3′
	Reverse: 5′-TCACCACAACGGGCAGAGAGGT-3′
TMEM109	Forward: 5′-ACACTGGATGCCTGGATTGGGC-3′
	Reverse: 5′-AAGCCGAGGAGCAGAGACAGCA-3′
KIAA1549	Forward: 5′-AGCGTGCCCTCCGTGTTCAT-3′
	Reverse: 5′-TGCCTCTGCTTGGCGGGATT-3′
UBE2I	Forward: 5′-TCCGTGGGAAGGAGGCTTGT-3′
	Reverse: 5′-TGGCTTGTGCTCGGACCCTT-3′
MAP3K3	Forward: 5′-ACGAATGTCCCGTGCCCAGA-3′
	Reverse: 5′-TTCCATAGCCCTCGCCGCTGAT-3′
YWHAH	Forward: 5′-CGCTATGAAGGCGGTGACAGAG-3′
	Reverse: 5′-AGGGTGAGGTTGTCTCGCAGCA-3′
ITGB8	Forward: 5′-GCCTCGTTCCTCTGGGCAGC-3′
	Reverse: 5′-TTCTGGACCCAGCGCAAGGC-3′

### Flow cytometry

When cells reached 70–80% confluence, they were washed with PBS, detached with 0.25% trypsin, and fixed with 75% ethanol overnight. After treatment with 1 mg/ml RNase A (Sigma) at 37°C for 30 min, cells were resuspended in 0.5 ml of PBS and stained with propidium iodide in the dark for 30 min. Fluorescence was measured with a FACScan flow cytometry system (BD Biosciences).

### mRNA stability assay

Experiments were carried out during logarithmic phase of cultured 293 cells. We treated 293 cell lines from time 0 with actinomycin D (10 μg/ml) for the indicated times. Then washed cultures in PBS, extracted RNA with RNA Extraction Kit, and analyzed RNA by quantitative RT-PCR (qRT-PCR).

### Data analysis

Microarray scan data were analyzed using the significance analysis of microarrays (SAM) R-package. To compare the results of different hybridization experiments, the signal intensity of each gene on different arrays was normalized versus the total intensity of all genes in the array. Corresponding normalized signals on different arrays were then compared to identify differential regulation in the gene expression between samples. Relative gene expression changes ≥1.5-fold were considered meaningful to represent up-regulation or down-regulation. Gene ontology (GO) analysis was performed to identify significantly enriched biological processes and molecular functions.

### Statistical analysis

All values are expressed as means ± SD in the figures. Statistical significance was assessed using Student’s *t*-test, and *P*-values of <0.05 were considered significant.

## Results

### Affymetrix cDNA microarray analysis of gene expression profiles following CSIG knockdown

**Figure 1:**
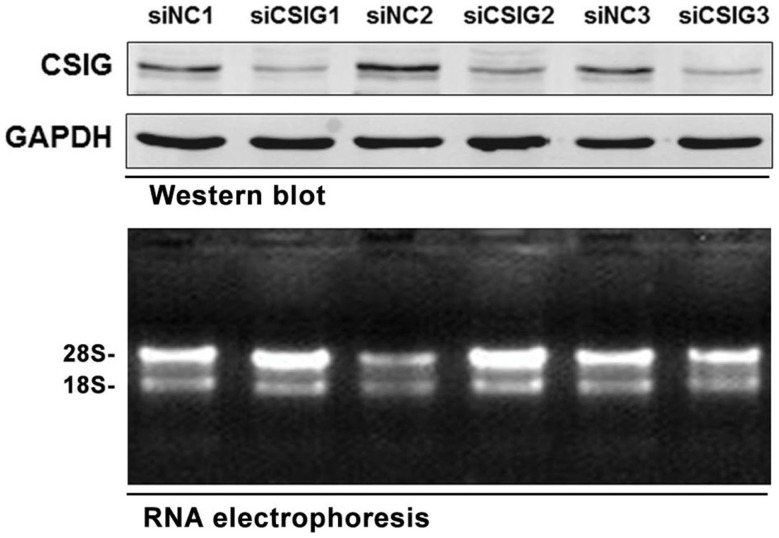
**The RNA samples prepared for Affymetrix microarray experiment**. Upper panel, western blot analysis of CSIG expression in siCSIG and siNC transiently transfected HEK293 cells. Total protein was extracted, and immunoblotting was performed using specific antibodies against CSIG as indicated. GAPDH served as a loading control. Bottom panel, the intactness of the RNA samples was tested using RNA electrophoresis. The three parallel experiments, indicated as 1, 2, and 3, respectively, were performed with the same siRNA.

To preparing RNA samples for microarray analysis, the small interfering RNA (siRNA) specifically targeting CSIG (siCSIG) and the control siRNA (siNC) targeting none of the human genes were transiently transfected into HEK293 cells, respectively, and cells were collected for analysis after 48 h. First, a small amount of samples were subject to western blot analysis of the gene knockdown efficiency. Comparing with control cells, CSIG siRNA transfection induced a more than 60% of decrease of CSIG level (Figure 1, upper panel). And then total RNAs from CSIG siRNA and control siRNA transfected cells were extracted from the remaining cultures in the same dish, respectively. Subsequently, two groups of samples in triplicate were subject to RNA integrity and purification examination. According to the RNA agarose gel analysis, the ratio of 28S:18S is 2:1 on the whole (Figure 1, bottom panel), which indicated the intactness of the RNA samples. And the ratio of A260:A280 is basically in the range of 1.7 ~ 2.0, which proved the purification of sample. Totally, the detection results indicated that the quality of RNA samples we prepared ultimately meets the requirements of Affymetrix cDNA microarray experiment.

**Figure 2:**
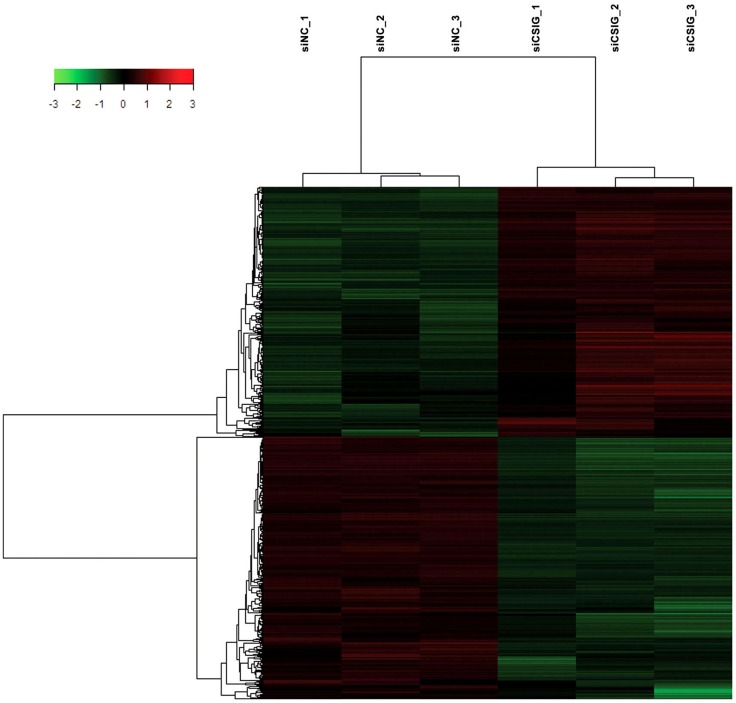
**Hierarchical clustering heat map of the 841 genes with significant differentially expressed changes following CSIG knockdown with *t*-test adjusted *P*-values **<**0.05, a fold-change cut off **≥**1.5, and FDR **<**5%**. Each column represents a sample; each row refers to a gene. Gene expression changes with respect to median changes are denoted by: red, up-regulated (ratio ≥1.5); green, down-regulated (ratio <1/1.5); and black, unchanged.

To screen important targets and signaling pathways modulated by CSIG, in this study, we analyzed the differential expression of genes in HEK293 cells from three CSIG knockdown and the corresponding control samples using six Affymetrix GeneChip Human Genome U133 plus 2.0 microarrays. The Affymetrix cDNA microarray analysis of the gene expression was performed as described in Section ‘Materials and Methods.’ The global genome gene expression profiles are analyzed and listed as mean values from triplicate GeneChips in supporting Table S1 in Supplementary Material. Differential expression analysis between CSIG knockdown and control cells was carried out with the SAM R-package software. We identified a total of 841 probe sets associated with 590 genes of known function – representing 4.7% of the 12487 well characterized human genes measurable on the microarray – that were expressed differentially between these two groups. The following criteria were used for gene selection: adjusted *P* < 0.05 and FC ≥1.5. Of these 590 genes, 311 (53%) were down-regulated and 279 (47%) were up-regulated (Figure 2). The majority of the selected genes showed moderate (yet significant) alterations in expression of between 1.5- and 2.0-fold (**Table** 2; for all genes, see Table S1 in Supplementary Material). Using adjusted *P* < 0.05 and FC ≥2 as a cutoff, there were totally 121 genes showing differential expression following CSIG knockdown, with 57 genes up-regulated (more than 2-folds increase) and 64 genes down-regulated (<0.5-folds decrease), respectively (**Table** 2).

**Table 2:** **The fold-change distribution of gene expression changes following CSIG knockdown**.

	FC **≥** 1.5	1.5 **≤** FC **<** 2	2 **≤** FC **<** 3	3 **≤** FC
Total genes	841	721	116	4
Up-regulated	411	355	55	2
Down-regulated	430	366	62	2
Percentage of FC genes in total genes	100	85.7	13.8	0.5

*The FC distribution of gene expression changes following CSIG knockdown. Shown is the number of genes in each FC size range for genes with FCs ≥1.5 and adjusted P-values <0.05; FC = fold change (detailed gene list, Table S1 in Supplementary Material)*.

**Figure 3:**
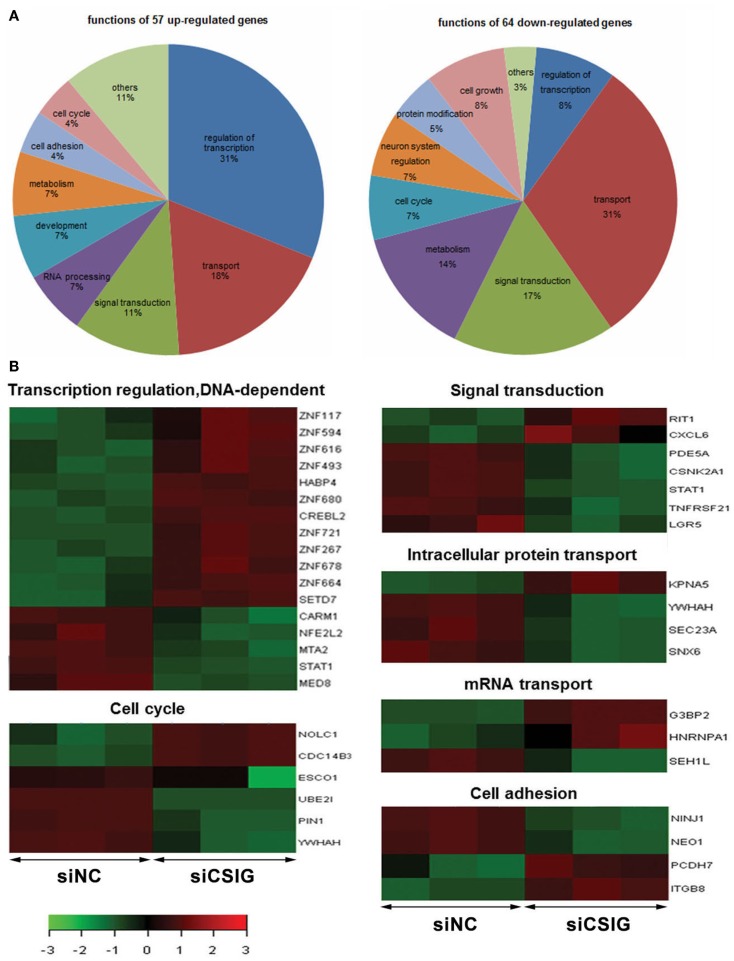
**Gene ontologies and functional analysis of the differentially expressed genes with *t*-test adjusted *P*-values **<**0.05, a fold-change cut off **≥**2**. **(A)** Gene ontologies with significantly over-represented differentially expressed genes following CSIG knockdown and the Ratio of genes implicated in various processes.**(B)** The differentially expressed genes implied in various functions by red–green hot spots. The color bar shows the fold change and corresponding color depth.

According to GO analysis and pathway analysis, the differentially expressed genes are implicated in a variety of process. By analysis using adjusted *P* < 0.05 and FC ≥2 as a cutoff, there are 31% of up-regulated genes implicated in regulation of transcription, and 31% of down-regulated genes involved in transport, respectively (Figure 3A). Notably, there are nine ZNF genes among them showing increased levels all together (Figure 3B). There are seven genes involved in signal transduction process with RIT1 and CXCL6 up-regulated while LGR5, PDE5A, CSNK2A1, STAT1, and TNFRSF21 down-regulated, six genes involved in cell cycle progress with NOLC1 and Cdc14B3 up-regulated while ESCO1, UBE2I, PIN1, and YWHAH down-regulated (Figure 3B). There are four genes participate in intracellular protein transport with KPNA5 up-regulated while YWHAH, SEC23A, and SNX6 down-regulated, and another four genes implicated in cell adhesion process with PCDH7 and ITGB8 up-regulated while NINJ1and NEO1 down-regulated (Figure 3B). There are three genes including G3BP2, HNRNPA1, and SEH1L involved in mRNA transport (Figure 3B).

### Validation of the differential expression of selected genes

**Figure 4:**
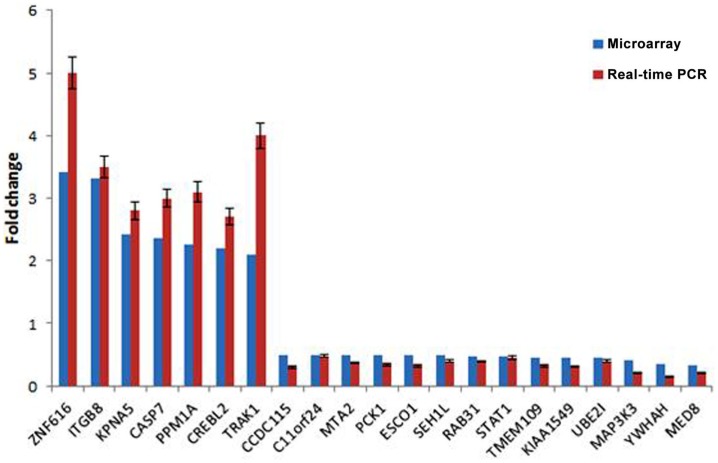
**Agreement between microarray and real-time quantitative RT-PCR data**. The blue block represent microarray data, the red block represent real-time PCR results. The results are mean ± SEM and the *P*-values are all <0.05.

To validate the result of Affymetrix microarray screen, we performed real-time qRT-PCR analysis in triplicate for 21 selected genes using cDNA from CSIG knockdown and control 293 cell samples. Specific primers were designed and synthesized as described in Section ‘Materials and Methods.’ The examined genes includes CCDC115, PPM1A, PCK1, KIAA1549, SETD7, ESCO1, CASP7, TMEM109, CREBL2, SEH1L, C11orf24, TRAK1, ZNF367, RAB31, STAT1, KPNA5, MAP3K3, NOLC1, HNRNPA1, YWHAH, ZNF616, MTA2, and UBE2I. CSIG siRNA and control siRNA were transiently transfected into HEK293 cells and cells were collected after 48 h for RNA extraction and reverse transcription. Consistent with the Affymetrix microarray data, real-time PCR result confirmed that the expressions of ZNF616, ITGB8, KPNA5, CASP7, PPM1A, CREBL2, and TRAK1 are increased (Figure 4), while expressions of CCDC115, C11orf24, MTA2, PCK1, ESCO1, SEH1L, RAB31, STAT1, TMEM109, KIAA1549, UBE2I, MAP3K3, YWHAH, and MED8 are decreased following CSIG knockdown, respectively (Figure 4). The CSIG-related changes in gene expression measured by qRT-PCR were in agreement with microarray data (Table 3). The microarrays thus provided a reliable comparison of gene expression in 293 cells following CSIG knockdown.

**Table 3:** **Verification of microarray data by quantitative real-time PCR**.

Symbol	Description	Fold (genechip)	Fold (qPCR)
ZNF616	Zinc finger protein 616	3.41	5.0
ITGB8	Integrin, beta 8	3.317	3.5
KPNA5	Karyopherin alpha 5 (importin alpha 6)	2.4291	2.8
CASP7	Caspase 7, apoptosis-related cysteine peptidase	2.3629	3.0
PPM1A	Protein phosphatase 1A (formerly 2C), magnesium-dependent, alpha isoform	2.2666	3.1
CREBL2	cAMP responsive element binding protein-like 2	2.1951	2.7
TRAK1	Trafficking protein, kinesin binding 1	2.0994	4.0
CCDC115	Coiled-coil domain-containing 115	0.4988	0.35
C11orf24	Chromosome 11 open reading frame 24	0.4952	0.48
MTA2	Metastasis associated 1 family, member 2	0.4949	0.4
PCK1	Phosphoenolpyruvate carboxykinase 1 (soluble)	0.49	0.36
ESCO1	Establishment of cohesion 1 homolog 1 (*S. cerevisiae*)	0.49	0.35
SEH1L	SEH1-like (*S. cerevisiae*)	0.4894	0.4
RAB31	RAB31, member RAS oncogene family	0.4715	0.4
STAT1	Signal transducer and activator of transcription 1, 91 kDa	0.4707	0.45
TMEM109	Transmembrane protein 109	0.456	0.35
KIAA1549	KIAA1549	0.45	0.35
UBE2I	Ubiquitin-conjugating enzyme E2I (UBC9 homolog, yeast)	0.4518	0.43
MAP3K3	Mitogen-activated protein kinase kinase kinase 3	0.4086	0.2
YWHAH	Tyrosine 3-monooxygenase/tryptophan 5-monooxygenase activation protein, eta polypeptide	0.3572	0.17
MED8	Mediator complex subunit 8	0.3653	0.25

**Figure 5:**
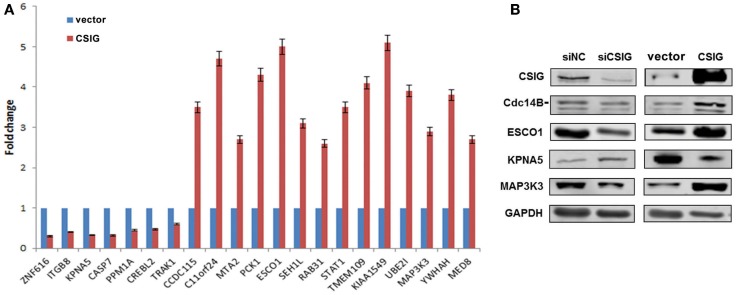
**The expressions of differentially expressed genes in CSIG overexpressed or silenced HEK293 cells**. **(A)** Real-time PCR analysis of gene expressions in CSIG-transfected and control HEK293 cells. The results are mean ± SEM and the *P*-values are all <0.05. **(B)** Western blot analysis of Cdc14B, ESCO1, KPNA5, and MAP3K3 expressions in HEK293 cells following CSIG knockdown or overexpression.

To further confirm the differential expression of above genes, we carried out real-time PCR analysis in CSIG-overexpressed HEK293 cells. On the contrary, following CSIG overexpression, the expressions of ZNF616, ITGB8, KPNA5, CASP7, PPM1A, CREBL2, and TRAK1 are decreased, while expressions of CCDC115, C11orf24, MTA2, PCK1, ESCO1, SEH1L, RAB31, STAT1, TMEM109, KIAA1549, UBE2I, MAP3K3, YWHAH, and MED8 are induced (Figure 5A). As predicted, by comparison with the expression patterns following CSIG-silencing, CSIG overexpression induced an inverse expression alterations, which validated the microarray data on the contrary. Western blot analysis of Cdc14B, ESCO1, KPNA5, and MAP3K3 was further carried out in CSIG-silencing and CSIG-overexpressed HEK293 cells. Following CSIG knockdown, Cdc14B, ESCO1, and MAP3K3 expressions are inhibited, while KPNA5 is increased (Figure 5B). Inversely, when CSIG overexpressed, Cdc14B, ESCO1, and MAP3K3 were induced, and KPNA5 is decreased correspondingly (Figure 5B). The observations are consistent with the real-time PCR results, indicated a positive expression correlation of Cdc14B, ESCO1, MAP3K3 with CSIG.

### Explore the correlation of differentially expressed genes with CSIG in senescence

**Figure 6:**
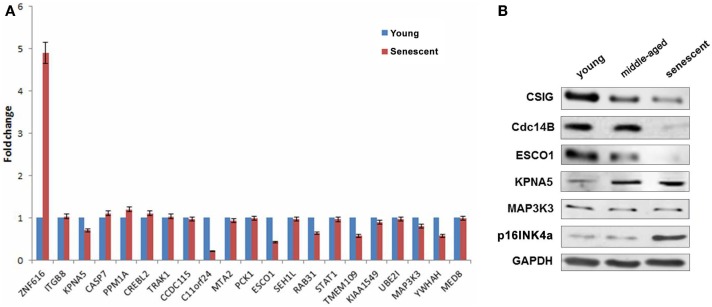
**Explore the correlation of differentially expressed genes with CSIG in senescence**. **(A)** Real-time PCR analysis of gene expressions in early-passaged (young) and senescent 2BS cells. The results are mean ± SEM and the *P*-values are all <0.05. **(B)** Western blot analysis of Cdc14B, ESCO1, KPNA5, and MAP3K3 expressions in early-passaged (young), middle-aged, and senescent 2BS cells. p16INK4a served as a senescence marker. And GAPDH served as negative control.

As described previously, CSIG is abundant in early-passage (young) cells but declined during senescence. To explore the correlation of differentially expressed genes with CSIG in senescence, the expressions of the selected 21 genes were detected by real-time PCR during senescence using the diploid fibroblast 2BS senescence model cells. As shown, comparing with the expression in young cells, ZNF616 was significantly induced in senescent cells while KPNA5, PPM1A, and CREBL2 were slightly increased (Figure 6A). In contrary, expressions of C11orf 24, ESCO1, TMEM109, and YWHAH were abundant in young cells, but decreased in senescent cells (Figure 6A). Furthermore, we analyzed the expressions of Cdc14B, ESCO1, KPNA5, and MAP3K3 in young (~18 PDs), middle-aged (~40 PDs), and senescent (~57 PDs) 2BS cells and found the decreased expressions of Cdc14B, ESCO1, and increased expressions of KPNA5 with the increasing PDs of 2BS cells, while no obvious changes for MAP3K3 (Figure 6B). The results suggested a CSIG-modulated expression pattern of these genes during senescence.

### Explore the correlation of differentially expressed genes with CSIG in cell cycle

**Figure 7:**
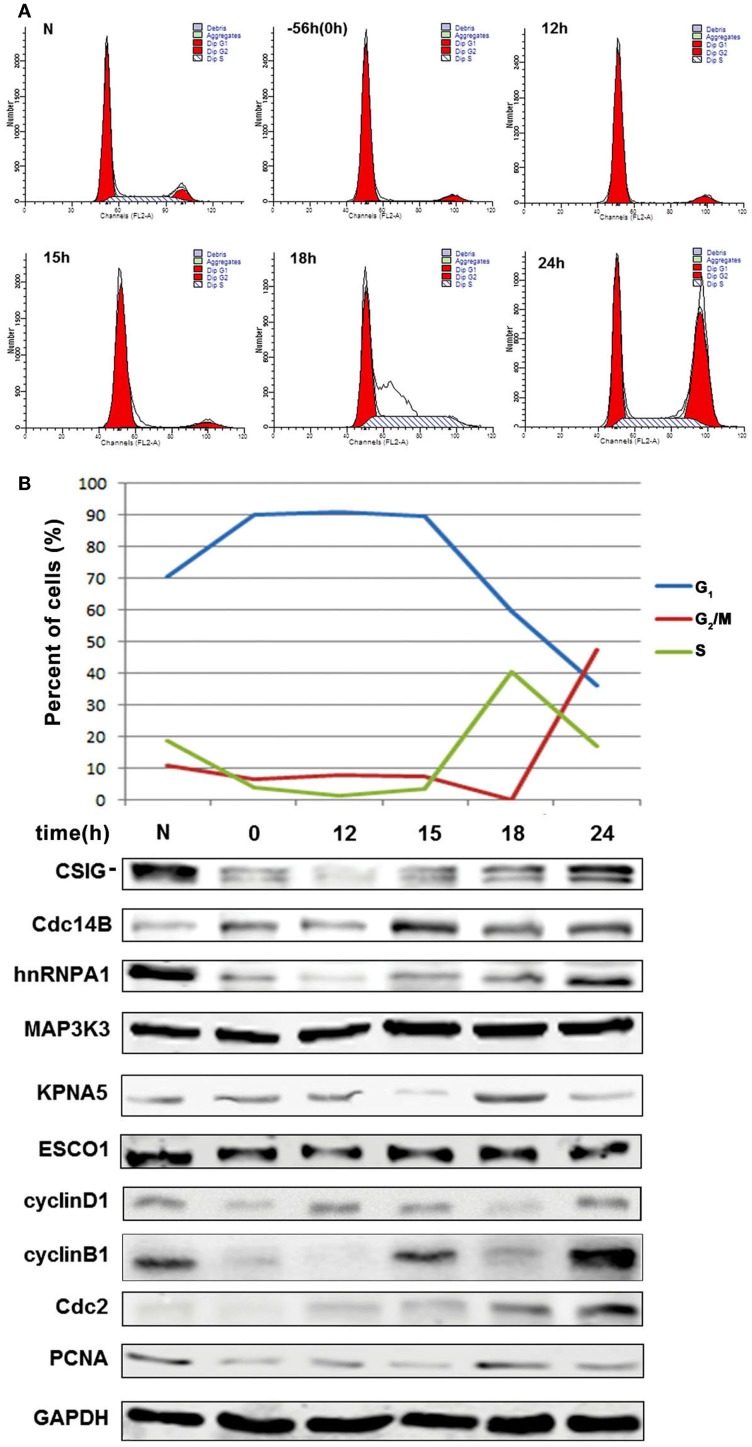
**Explore the correlation of differentially expressed genes with CSIG in cell cycle**. **(A)** Flow cytometry analysis of cell cycle phases following cell synchronization. Early-passaged 2BS cells (18 PDs) were serum-starved for 56 h and then cultured in normal medium with 10% FBS. Cells were collected at different time points for cell cycle analysis by flow cytometry. **(B)** Upper panel, Sketch map of cell cycle phases (G_1_, G_2_/M, and S phase) according to values of cell cycle distribution at different time points. Bottom panel, western blot analysis of the expressions of CSIG, Cdc14B, hnRNPA1, MAP3K3, KPNA5, and ESCO1 during cell cycle. N represents normal 2BS cells. 0 represents a time point when cells starved for 56 h. cyclinD1, cyclinB1, Cdc2, and PCNA served as positive control (cell cycle phase marker proteins). And GAPDH served as negative control.

As GO and pathway analysis showed that several differentially expressed genes were implicated in cell cycle progression. It will be of great interest to study whether CSIG regulate senescence through regulating cell cycle-associated proteins. To explore the correlation of differentially expressed genes with CSIG in cell cycle, we examined the expression patterns of them during cell cycle phases. Young 2BS cells were synchronized by serum starvation for 56 h and then regained to culture with DMEM containing 10% FBS. Cells were collected at different time points for flow cytometry analysis and western blot analysis. According to cell cycle distribution shown in Figure 7A, following serum starvation treatment, more than 90% cells were synchronized at G_1_ phase (Figure 7A). When recovered by addition of 10% FBS, cells reentered into normal cell cycle process gradually. The percent of S phase cells reached to the highest 40.58% at 18 h following recovery, while the percent of cells in G_2_/M phase is nearly 0 (Figure 7A). At 24 h point, the percent of G_2_/M phase cells was increased to 47.36% with very low percent of S and G1 phase cells, accordingly (Figure 7A).

As known, cyclin D1 is accumulated in G_1_ phase, cyclin B1, and Cdc2 in transition of S phase to G_2_/M phase and G_2_/M phase, and PCNA in early G_1_ phase and S phase (Figure 7B). Thus, we simultaneously detected the expressions of cell cycle proteins as the positive control for specific cell cycle phases. Western blot analysis showed that CSIG expression is fluctuated during cell cycle and accumulates in S phase (~18 h) and G_2_/M phase (~24 h) of cell cycle, and Cdc14B is abundant in G_1_ phase, S phase (~18 h) and G_2_/M phase (~24 h), hnRNP A1 is induced in G_2_/M phase (~24 h), and KPNA5 in S phase (~18 h), respectively (Figure 7B). Meanwhile, we observed no expression alteration of ESCO1 and MAP3K3 during cell cycle (Figure 7B).

### CSIG modulates CREBL2, caspase7, and Cdc14B mRNA turnover

**Figure 8:**
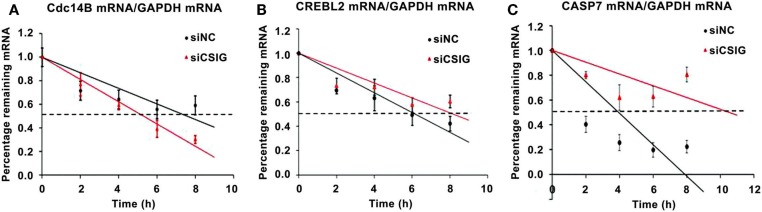
**CSIG regulates the stability of Cdc14B, CREBL2, and Caspase7 mRNA in HEK293 cells**. RNA was isolated at the indicated times after actinomycin D application to HEK293 cell lines, and the stability of Cdc14B, CREBL2, and Caspase7(CASP7) mRNA was normalized to the values for GAPDH mRNA. **(A)** Cdc14B mRNA half-life is shortened following CSIG knockdown. **(B)** CREBL2 mRNA half-life is prolonged following CSIG knockdown. **(C)** CASP7 mRNA half-life is prolonged following CSIG knockdown.

As a RNA-binding protein previously reported by our and other labs we supposed that CSIG might mainly play a role on post-transcriptional level (7, 15), affecting mRNA degradation or translation. To detect whether the CSIG-associated mRNA level alterations are resulted from the mRNA degradation, we performed the pulse-chase experiment to examine the half-life of several selected mRNA. As shown in Figure 8, following CSIG knockdown, the half-life of Cdc14B mRNA is shortened by ~2 h (Figure 8A), while the half-life of CREBL2 and Caspase7 mRNA is prolonged by 2 and 6.5 h, respectively (Figure 8B,C), indicating CSIG implication in mRNA turnover.

## Discussion

The present study stemmed from our previous findings. Using a suppressive subtractive hybridization system, we previously identified a CSIG (also known as RSL1D1), which is involved in important processes including senescence, cell cycle regulation, stress response, and tumor metastasis. Here, we set out to explore the regulatory mechanism underlying. To screen important targets and signaling pathways modulated by CSIG, we investigated the differential gene expression profiles following CSIG knockdown in HEK293 cells using the Affymetrix GeneChip microarray. Of the 12,487 genes represented on the microarray, 279 genes, including ZNF616, ITGB8, CASP7, and PPM1A, were up-regulated and 311 genes, including STAT1, UBE2I, MAP3K3, MED8, PCK1, and ESCO1, were down-regulated in CSIG knockdown 293 cells. The differentially expressed genes are involved in a broad array of biological processes, mainly in cell cycle, signal transduction, transcriptional regulation, development, and cell adhesion (Figure 3), which offers the possibility of CSIG participation in these processes. Notably, according to GO and pathway analysis, 17 of the changed genes including 9 ZNF genes were implicated in DNA-dependent transcription regulation. Among them, ZNF616 showed the most significant increase following CSIG knockdown. Consistent to the senescence-inhibited expression of CSIG, we observed the senescence-induced expression (low in young cells, while increased in senescent cells) of ZNF616, which suggested the potential CSIG–ZNF616 pathway during senescence progression.

The differential expression of genes such as ZNF616, ESCO1, KPNA5, and MAP3K3 was further validated by real-time PCR and western blot analysis. We further demonstrate the correlated expression patterns of ESCO1, KPNA5, and MAP3K3 with CSIG during cell cycle and senescence progression. According to real-time PCR analysis, ZNF616, c11orf24, ESCO1, and YWHAH exhibit differential expression during senescence. Furthermore, western blot analysis showed a cell age-dependent expression of Cdc14B, ESCO1, and KPNA5 (Figure 6). Cell division cycle 14B (Cdc14B), a bidirectional phosphatase, is involved in cell cycle (yeast), DNA damage response, DNA repair, and aging process (16–18). Establishment of cohesion 1 homolog 1 (ESCO1), belonging to a conserved family of acetyltransferases (19, 20), is mainly involved in sister chromatid cohesion (21) and DNA damage repair (22). KPNA5 belongs to the importin α protein family and is thought to be involved in nuclear localization signals (NLS)-dependent protein import into the nucleus (23). The expression and function correlations with CSIG suggested that these proteins might act as potential downstream effectors or mediators of CSIG to regulating cell proliferation and senescence. Further studies on link between CSIG-regulated genes and senescence will be a significant work. Recently, we observed one of CSIG-regulated genes, Cdc14B, really modulates senescence progression (unpublished). It is likely that CSIG is also involved in the regulation of *in vivo* aging. In this regard, studies to develop a knock-out system and to further validate the cellular targets of CSIG during tissue aging are underway in our laboratory. The results of these studies will hopefully provide a more complete understanding of the role of CSIG and its mechanisms of action.

Our previous results showed that CSIG predominantly localized in the nucleolus, the major site for synthesizing and assembling ribosomal subunits. As a nucleolus protein, CSIG is expressed extensively and abundantly in cells. And it has been proved by our practical work that the effect of CSIG knockdown is better than CSIG overexpression. Therefore, we adopted the gene knockdown strategy. According to our previous study (7) and report (15), CSIG may be an mRNA-binding protein, which means that CSIG might regulate gene expression mainly on post-transcriptional level (mRNA turnover and translation). Therefore, we examined the mRNA half-life change following CSIG knockdown. Consequently, our observations proved the presumption, and the mechanism exploring is under way in our lab.

Together, the analysis of differentially expressed genes following CSIG knockdown provides important clues for the regulatory mechanisms of CSIG in proliferation and senescence. This differential expression profile in response to CSIG knockdown should prove useful for identification of target genes and for elucidating the molecular mechanism responsible for the regulation of cell proliferation and senescence by CSIG.

## Conflict of Interest Statement

The authors declare that the research was conducted in the absence of any commercial or financial relationships that could be construed as a potential conflict of interest.

## Supplementary Material

The Supplementary Material for this article can be found online at http://journal.frontiersin.org/article/10.3389/fendo.2015.00069/abstract
